# Retreatment of oval-shaped root canals filled with TotalFill bioceramic or AH plus sealer

**DOI:** 10.1038/s41598-023-36608-0

**Published:** 2023-06-08

**Authors:** Ahmed Jamleh, Mohannad Nassar, Abdulmohsen Alfadley, Azhar Alanazi, Hadeel Alotiabi, Maryam Alghilan, Khalid Alfouzan

**Affiliations:** 1 Restorative and Prosthetic Dental Sciences, College of Dentistry, King Saud bin Abdulaziz University for Health Sciences, National Guard Health Affairs, P.O. Box 22490, 11426 Riyadh, Kingdom of Saudi Arabia; 2 King Abdullah International Medical Research Centre, National Guard Health Affairs, Riyadh, Kingdom of Saudi Arabia; 3 Department of Preventive and Restorative Dentistry, College of Dental Medicine, University of Sharjah, Sharjah, United Arab Emirates; 4 Endodontic Division, Dental Services, Central Region, King Abdulaziz Medical City, Ministry of National Guard Health Affairs, Riyadh, Saudi Arabia

**Keywords:** Biomaterials, Cone-beam computed tomography, Endodontic files

## Abstract

This study investigated retreatment of oval canals filled with gutta-percha and different sealers using WaveOne Gold (WOG). Single oval canals were prepared to size 30, 0.04 and obturated with gutta percha and AH Plus (AHP) or TotalFill bioceramic (TFBC) sealer. After 6-month incubation, the canals were retreated with WOG Primary (25, 0.07) under simulated body temperature, and the developed load and torque were simultaneously measured. The time and regaining the apical patency were checked. Micro-computed tomography scanning was performed to calculate the remaining obturating materials. An independent t-test and chi-square test were performed at a 95% confidence level. A shorter retreatment time was needed in TFBC than in AHP (*P* = 0.003). However, a higher maximum apical load was reported with AHP (*P* = 0.000). Meanwhile, comparable maximum coronal load and maximum torque values were observed. Apical patency was regained in all TFBC roots and only 75% of the AHP samples (*P* = 0.217). The remaining obturating materials were comparable in TFBC (13.02 ± 8.12%) and AHP (10.11 ± 8.46%) (*P* = 0.398). WOG was able to remove 89.89% and 86.98% of obturating materials in TFBC and AHP, respectively. The TFBC presented lower apical loads and faster retreatment compared to AHP.

## Introduction

Nonsurgical retreatment is a procedure that involves the removal of obturating materials to eradicate persistent bacteria and provide potential space for efficient root canal system disinfection and re-obturation^1^. This modality is considered successful through addressing the present pathologic conditions and/or iatrogenic errors and preventing their occurrence during the procedure. Compared to initial treatment, the retreatment procedure is considered more challenging as it might compromise more steps such as the removal of coronal restorations, allocating missing canals, removing obturating materials, regaining the original canal path, repairing perforations, and removing endodontic posts and separated files^1–3^. The removal of obturating materials imposes more stress on the files and the canal walls. This is evident in a previous study which revealed that NiTi files fractured more in retreatment cases compared to initial treatment^4^.

Canal obturation is usually performed with gutta-percha as the core material along with an endodontic sealer. Endodontic sealers can be classified based on their chemical formula. AH Plus sealer (AHP) (Dentsply, Tulsa, OK, USA), an epoxy resin-based sealer, shows good sealing and bonding abilities between dentin and gutta-percha^5^. Hydraulic calcium silicate-based sealer (HCSS) sealers have been increasingly adopted in the market as they exhibit excellent properties with promising outcomes^6,7^. HCSS sealers become hard on setting and has the ability to penetrate the dentinal tubules and form hydroxyapatite crystals on the interface with dentin^8^. TotalFill bioceramic sealer (TFBC) (FKG Dentaire SA, La Chaux-de-Fonds, Switzerland), a premixed HCSS sealer, was shown to be as effective as AHP in terms of the sealing ability when used with gutta-percha in a matched cone obturation technique^9^.

WaveOne Gold (WOG, Dentsply Sirona) is a new version of the WaveOne system. It has a different geometry and is made of gold wire. These differences resulted in improved mechanical properties and performance of WOG compared with WaveOne^10–12^. Furthermore, WOG was found to be efficient in removing the obturating materials during the retreatment procedure^13,14^.

Measuring vertical load and torque might give insights into how sealers influence endodontic root canal retreatment. Several studies have investigated the vertical load and torque induced during initial root canal treatment. In a clinical setting, dentinal damage might take place due to stresses and torques generated on the walls during canal preparation mainly close to the file tip^15^. However, limited number of studies measured these parameters during retreatment^16–19^. Therefore, the current study’s purpose is to investigate the effect of TFBC and AHP on the removal of obturating materials from root canals with the use of WOG. The null hypothesis was that AHP and TFBC have similar outcomes in terms of the percentage of remaining obturating materials and the generated retreatment load and torque.

## Materials and methods

### Teeth selection

The protocol of this study was approved by an institutional review board from King Abdullah International Medical Research Center (RC20/570/R). All methods were performed in compliance with the relevant guidelines and regulations.

Intact permanent premolar teeth with single canals were selected and stored in distilled water. The teeth were extracted for unknown reasons after obtaining informed consent from patients. They were inspected under a dental operating microscope at 4 × magnification (OPMI Zeiss Pico; Carl Zeiss MediTec, Dublin, CA, USA) to detect root surface defects. They were radiographically observed in buccolingual and mesiodistal aspects. Teeth with oval straight canals were included if the canal diameter in the buccolingual aspect was at least twice larger than the mesiodistal diameter at any level in the coronal two-thirds of the canal. Teeth with curved canals, incomplete roots, root cracks, wide apex, or root canal filling were excluded.

Based on a pilot study and using a two-sample t-test, the sample size was calculated (PiFace, http://homepage.stat.uiowa.edu/~rlenth/Power/) with an 80% power and 5% significance level. The minimum mean differences (standard deviations) of the retreatment time, maximum load, maximum torque, and remaining obturating materials percentage were set found to be 9 (6), 0.5 (0.4), 0.4 (0.3), and 7 (5), respectively. According to  these parameters, a minimum number of eleven samples per each group was required.

### Sample preparation

The tooth root was covered with aluminum foil which was then immersed in a mixed resin (Duralay; Reliance Dental Mfg, Worth, IL, USA) inside a 12-mm height plastic transparent tube. After the resin set, the foil was removed and a light-body silicone impression material was added to simulate the periodontal ligament. Access cavity was created and a size 10 K-file file (SybronEndo, Orange, CA, USA) was used to verify canal patency and set the working length short of the foramen by 0.5 mm. The manual glide path was made up to size 15 K-file. Then, canals were prepared with HyFlex CM rotary file (Coltène Whaledent, Altstätten, Switzerland) to size 30, 0.04. Canal patency was maintained through recapitulation and irrigation with 5 ml of 2.5% sodium hypochlorite (NaOCl) throughout the canal preparation. Finally, the canals were irrigated with 2 ml of 17% EDTA and flushed with 2 ml of 0.9% sodium chloride. A 27G side-vented irrigating needle was used during the irrigation step.

The prepared canals were coded and distributed randomly (using www.random.org) into two groups of 12 samples based on the endodontic sealer used: AHP and TFBC.

### Canal obturation

Each canal was obturated with a matched gutta-percha cone size 30, 0.04 according to manufacturers’ instructions. The canals were dried completely with matching paper points.

For the AHP group, the sealer was placed with a sterilized K-file. Also, the cone was coated with the sealer and inserted to the working length. For the TFBC group, the sealer was injected after positioning the syringe tip in the root canal. Then, the cone was coated with TFBC and inserted to the working length.

The excess gutta-percha was cut using B&L SuperEndo Alpha II unit (B&L BioTech, Philadelphia, PA, USA) at a level below the orifice. Then, the samples were coronally temporized with Coltosol F (Coltène Whaledent, Altstätten, Switzerland), and periapical radiographs were obtained to check the quality of the obturation. In case of unsatisfactory obturation quality, the sample was discarded and replaced. Then, the samples were placed in a test tube containing phosphate-buffered saline (PBS) and incubated for 6 months at 37 °C and 100% humidity. Canal shaping and obturation were performed by a single operator (Az. A).

### Micro-computed tomography (micro-CT) examination

Each sample was scanned twice (before and after the retreatment procedure) using a micro-CT device (SkyScan 1172; Bruker micro-CT, Kontich, Belgium) with the following parameters: 70 kV, 139 μA, aluminum filter with 0.5 mm thickness, voxel size of 13.6 μm, rotational step of 0.80-degree, rotational angle of 180-degree, exposure time of 2 s, and 3X frame averaging.

The resultant JPG slices were used to create 3D reconstruction using NRecon software (Bruker micro-CT) with 10X ring artifact correction and 50% beam hardening correction.

CTAn software (Bruker micro-CT) was used to perform segmentation and thresholding for the apical 12 mm of the sample to measure the volumes of obturating materials before and after retreatment. The percentage of the remaining obturating material was calculated by dividing the volume of the remaining obturating material after retreatment by the volume of the obturating material before retreatment and multiplying the result by 100.

### Canal retreatment

The coronal 2 mm of obturating materials were removed using a size 3 Gates Glidden drill (Dentsply Sirona, Ballaigues, Switzerland). Then, 1–2 drops of chloroform were used to soften the obturating material to facilitate file penetration. WOG Primary file (size 25, 0.07) was operated with reciprocating motion using the “WAVEONE ALL” mode in the X-Smart Plus motor (Dentsply Sirona) to remove the obturating materials. Attempts were made to reach the working length. Then, 10 vertical brushing movements with 3 mm amplitude were performed. Afterward, the canal was dried with paper points and checked for remnants of gutta-percha under the microscope at 8 × magnification. When gutta-percha was found on the canal walls, another 10 vertical strokes were performed. The canals were irrigated using a 27G side-vented irrigating needle with a total of 9 ml volume (5 ml of 2.5% NaOCl throughout the procedure and a final rinse of 2 ml of 17% EDTA was performed before flushing with 2 ml of 0.9% sodium chloride). The total amount of time needed for retreatment by the use of the WOG Primary file inside the canal, including the additional strokes, where applicable, and irrigation was calculated. Regaining the apical patency was inspected using a size 15 K-file.

To mimic the clinical conditions, the procedure was conducted while the sample was surrounded with warm water at 35 ± 1 °C.

### Load and torque measurements

**Figure 1:**
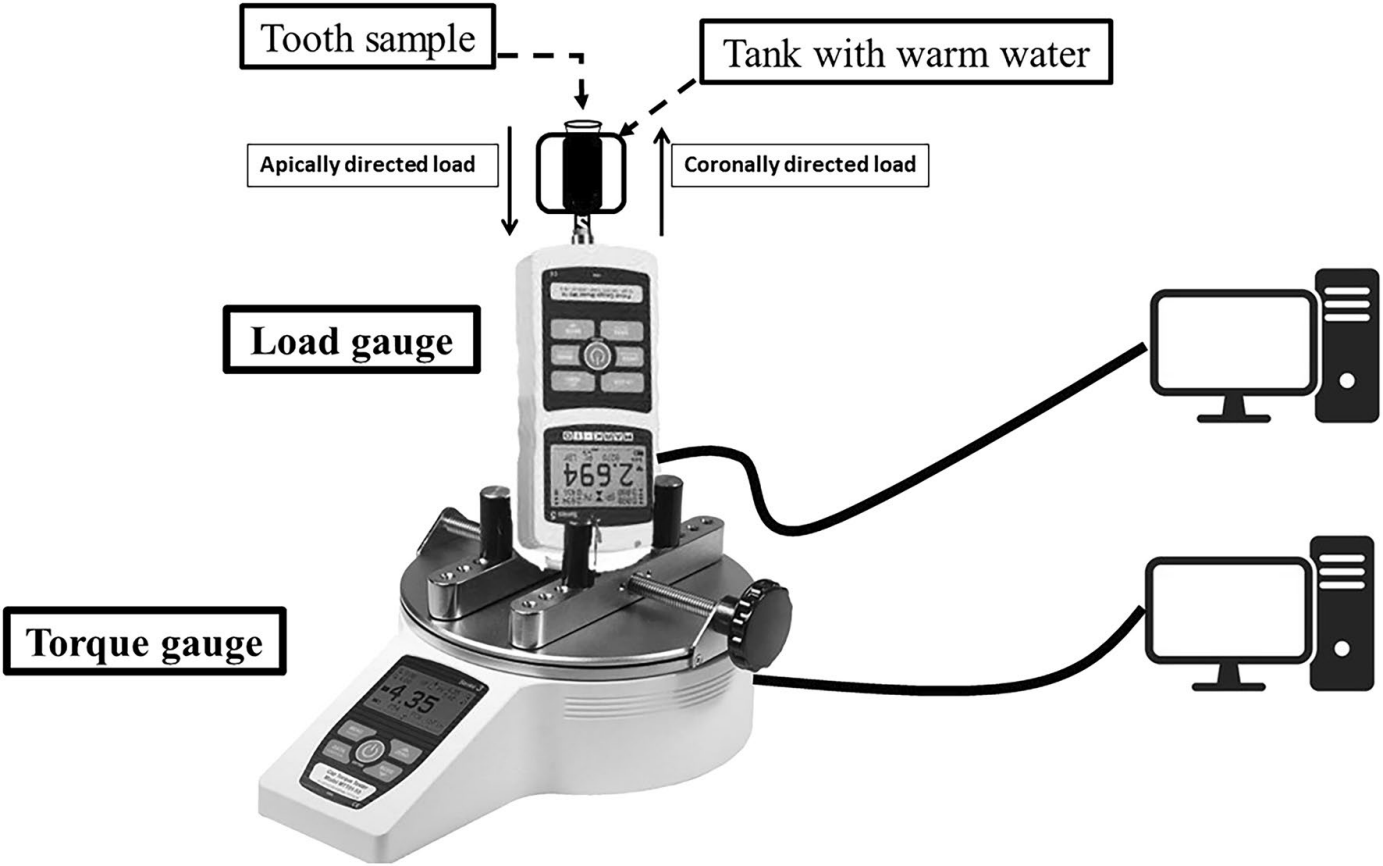
Schematic drawing of the experimental set-up.

A load gauge (M5-20 Advanced Digital Force Gauge; Mark- 10 Corporation) and a torque gauge (TT01 torque Gauge; Mark-10 Corporation) were used in the current study and were checked for accuracy before the retreatment. The load gauge was centered and fixed in a standing position above the torque gauge. Then, each sample was secured at the top of the load gauge. With this, measuring the developed real-time vertical load and torque simultaneously using MESUR Lite software (Mark-10 Corporation, Copiague, NY, USA) was possible (Fig. 1). The apically directed load represents the positive load required to advance the file inside the canal. The coronally directed load represents the load required to remove the file from the canal against resistance. The positive torque values were observed. The gauge devices measured data every 0.1 s.

### Statistical analysis

Statistical analyses were performed using the statistical program SPSS version 22 (IBM, Chicago, IL, USA) at a 95% confidence level. Since the distribution of the tested variables data was normal by the Kolmogorov–Smirnov test (*P* > 0.05), the independent t-test test was used to compare the experimental groups in terms of the effective retreatment time, the maximum load in apical and coronal directions, the maximum torque, and the percentage of remaining obturating materials. The number of teeth where apical patency was regained in both groups was compared by employing the chi-square test.

## Results

The mean and standard deviation data of the effective retreatment time, maximum retreatment loads and torque, and percentage of remaining obturating materials for the AHP and TFBC groups are presented in Table 1.

**Table 1:** Descriptive data (Mean ± standard deviation) of the retreatment time, maximum retreatment loads and torques, and remaining obturating materials in each group.

	Before volume (mm3)	Retreatment Time (sec)	Maximum Apical Load (N)	Maximum Coronal Load (N)	Maximum Torque (Ncm)	Remaining Obturating Materials (%)	Additional Strokes	Regain Apical Patency
AH Plus	5.90 ± 1.53	113.09 ± 33.10	1.50 ± 0.40	0.17 ± 0.04	1.24 ± 0.56	10.11 ± 8.46	0/12	9/12
TotalFill Bioceramic	7.36 ± 2.60	75.92 ± 20.93	0.71 ± 0.23	0.21 ± 0.07	1.43 ± 1.06	13.02 ± 8.12	5/12	12/12
p-value	0.108	0.003	0.000	0.140	0.588	0.398	0.037	0.217

A shorter retreatment time was needed in TFBC than in AHP (*P* = 0.003). The maximum apical load in AHP was higher than that in TFBC (*P* = 0.000). Both groups revealed similar maximum coronal load and maximum torque values (*P* > 0.05).

**Figure 2:**
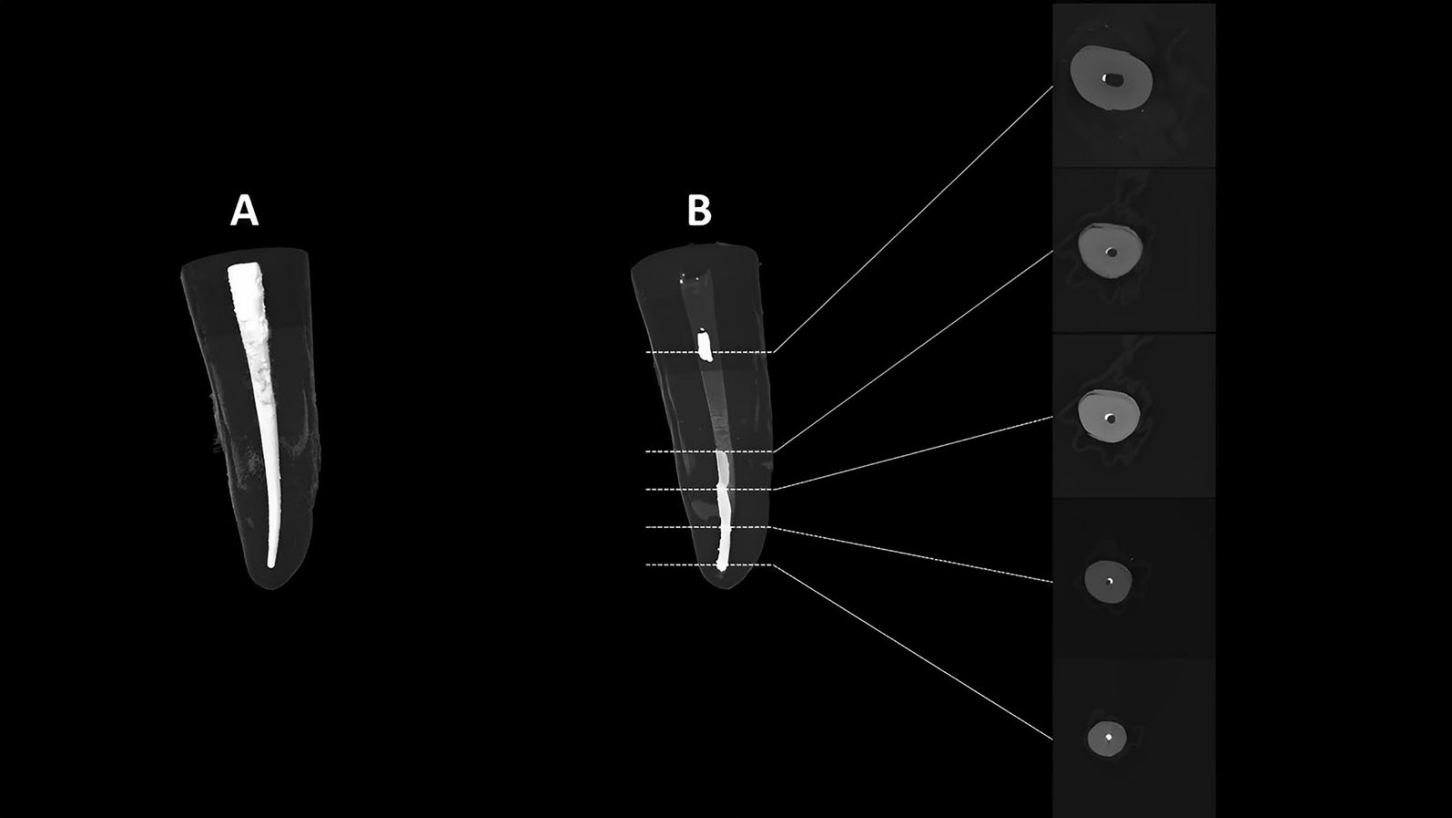
Representative microCT images of a sample taken before (**A**) and after retreatment with WaveOne Gold Primary (**B**) to calculate the percentage of remaining obturating materials. Note that the majority of the remaining obturating materials were located on the buccal/lingual walls and at the apical area. Apical patency was not regained in this sample, and this finding was observed only in three AH Plus samples.

The volumes of obturating materials measured before and after retreatment were used to calculate the remaining obturating materials percentage (Fig. 2). The mean percentages were found comparable between TFBC and AHP (*P* = 0.398). Apical patency was regained in all TFBC roots and only 75% of the AHP samples (*P* = 0.217).

## Discussion

The ability to remove the obturating materials and explore areas containing infected tissues is an important stage for the success of endodontic retreatment^20^. The current study investigated the endodontic retreatment of root canals filled with gutta-percha and TFBC or AHP sealer by using the WOG. In this study, there were no differences between the used endodontic sealers in terms of maximum coronal load, maximum torque, and remaining obturating materials. However, the retreatment time and maximum apical load in TFBC were lower than in AHP. Thus, the null hypothesis was partially accepted.

It should be highlighted that the root canals were initially prepared with a HyFlex CM 30/0.04 and then obturated with a matched gutta-percha cone. The retreatment procedure was performed using a WOG 25/0.07. In this study, our aim was to assess the ability of WOG to remove gutta-percha in oval canals without additional shaping. Thus, in order to compensate for differences in the size and taper of the used HyFlex and WOG, brush-stroke motions were used during retrieval of the obturating materials. The WOG was able to remove more than 86% of the obturating materials in the tested groups. The bulk of the published literature on orthograde retreatment is in agreement that complete removal of obturating materials is not achievable regardless of the file system used^19–30^. Hence, consistent with past studies, the WOG is considered a reliable single-file system for retreatment purpose^13,14^. In general, the retreatment procedure is influenced by many factors such as canal geometry, preparation size, file system, motion kinematics, and the type of obturating materials^20–30^.

In this study, these factors were standardized except for the endodontic sealer type. The sealer type was shown to influence the generated load where roots obturated with TFBC demonstrated lower apical loads when compared with those obturated with AHP. Moreover, TFBC-obturated root canals required less retreatment time than those obturated with AHP, which corroborates previous findings.^19,23^ The obturating materials would result in more pressure on the file and canal walls during retreatment which necessitates the application of higher load and torque. A previous retreatment study showed that the XP Shaper generated apical loads that reached 2.62 N^18^. However, the WOG generated lesser retreatment apical loads ranging from 0.71 to 1.50 N and required more time to retreat the tested groups. This could be attributed to differences between the tested systems in the file tip size (25 vs. 27), motion kinematics (Reciprocating Vs Continuous), rotational speed (350 rpm vs. 3000 rpm), and file geometry.

The introduction of new obturating materials into the market such as HCSS sealers with the increased adoption calls for a need to investigate their retreatability. In clinical practice, low-quality root canal obturation and/or post-endodontic restorations may result in failure within months or years after initial treatment^31^. During this period changes in the obturation characteristics might arise such as an increase in voids in root canals obturated with HCSS sealers^32,33^. These alterations may complicate or facilitate the retreatment procedure. Thus, to simulate a clinical scenario and to consider the changes in the characteristics of obturating materials over time, the tested samples in this study were retreated after 6 months of aging.

Five TFBC samples had clinically visible remaining gutta-percha on the canal walls after the initial 10 brushing strokes, and thus additional strokes were performed as suggested by a previous retreatment study^34^. In the current investigation, it is possible that TFBC could have benefited from these additional strokes that had led to a comparable amount of remaining obturating materials between the groups. However, this finding calls attention to the need for further research on the retreatment protocol for root canals obturated with bioceramic sealers.

Although TFBC and AHP had comparable remaining obturating materials, the apical load and retreatment time for TFBC was significantly lower. These findings corroborate published studies that found that HCSS sealers might be easily flaked off from root canals^23–27,30^. This might be explained by the sealer’s bond strength to dentin and its solubility characteristics. Indeed, the push-out bond strength of HCSS sealers is inferior when compared to AHP^35–37^. Solubility is another important property since it may compromise the overall quality of the root canal treatment leading to apical leakage. A meta-analysis revealed higher solubility of TFBC compared to the AHP because of the unpredictable setting of the HCSS sealer and the hydrophilic HCSS particles that enhance liquid absorption over time^38^. However, the dentin bonding mechanism of HCSS sealers upon setting is not fully addressed and further investigations to achieve better understating of the characteristics of HCSS sealers are required^39^. Under the present study conditions, the findings indicate easier and more predictable retreatment of root canals obturated with TFBC compared with AHP.

The ability to achieve apical patency for efficient disinfection close to the canal exit is one of the prognostic factors for apical tissue healing^40^. In this experiment, apical patency was achieved in all TFBC teeth and only 75% of the AHP teeth. Consistently, previous studies have reported that apical patency was achieved in 100% of samples with HCSS sealers^19,24,29^. Meanwhile, Hess et al. found that apical patency was re-established in 80% of samples with HCSS sealer despite regaining the full WL in all of them. In their study, they used the mesiobuccal root of mandibular molars with curvature less than 20º with no clear method of maintaining patency during the initial treatment. They elaborated that the remaining sealer in the apical foramen prevented the re-establishment of patency in 20% of the samples^21^. However, in our study straight oval-shaped canals of premolar teeth were utilized and canal patency was maintained during the initial treatment. Moreover, the WL was estimated at 0.5 mm from the exit of the canal in our study compared to 1 mm in Hess et al. Collectively, we believe that the methodology setup in the current study facilitated the removal of the sealer from the apical portion of the used canals. Thus, the aforementioned differences between the two studies might have resulted in the observed slight discrepancy in the number of canals with regained canal patency after retreatment.

The remaining materials were mostly located on the buccal and lingual walls. This can be explained by the inclusion of oval canals. This anatomical variation is clinically considered a challenge to the complete removal of obturating materials even with the use of the XP Shaper file that is designed to contact the majority of canal surfaces^19^.

The current results should be interpreted with caution since there are certain limitations to be considered. The utilization of extracted teeth from different patients and the use of in vitro experimental conditions can provide only partial answers to more comprehensive problems. Great care was strictly taken to balance all variables to merely study the influence of endodontic sealers on endodontic retreatment. All the tested teeth had single oval canals and were distributed equally based on the volume of obturating materials (*P* > 0.05). The retreatment procedure was conducted by an endodontist who is trained to use WOG in retreatment and was not aware of the type of sealer used in each sample. The procedure was performed in gentle strokes with in-and-out movements to keep steady pressure on the file. Moreover, the region of interest was limited to the apical 12 mm to eliminate the coronal area instrumented by the Gates Glidden drill.

Differences in the remaining obturating materials, time, and generated loads and torques during retreatment could have clinical relevance. However, the present study tested them in 2 sealers with one file system. This should be further investigated with other NiTi file systems devoted to removing obturating materials with other endodontic sealers.

## Conclusions

Under the conditions of this study, WOG was able to remove 89.89% and 86.98% of obturating materials in TFBC and AHP, respectively, in extracted teeth with oval canals. The TFBC presented lower apical loads and faster endodontic retreatment compared with the AHP.

## Data Availability

The numerical data generated in this work are available from the corresponding author upon reasonable request.
